# Real-Time Human Activity Recognition with IMU and Encoder Sensors in Wearable Exoskeleton Robot via Deep Learning Networks

**DOI:** 10.3390/s22249690

**Published:** 2022-12-10

**Authors:** Ismael Espinoza Jaramillo, Jin Gyun Jeong, Patricio Rivera Lopez, Choong-Ho Lee, Do-Yeon Kang, Tae-Jun Ha, Ji-Heon Oh, Hwanseok Jung, Jin Hyuk Lee, Won Hee Lee, Tae-Seong Kim

**Affiliations:** 1 Department of Electronics and Information Convergence Engineering, Kyung Hee University, Yongin 17104, Republic of Korea; 2 AI Laboratory, DeltaX, Seoul 04522, Republic of Korea; 3 Hyundai Rotem, Uiwang-si 16082, Republic of Korea; 4 Department of Software Convergence, Kyung Hee University, Yongin 17104, Republic of Korea

**Keywords:** real-time human activity recognition, deep learning networks, wearable exoskeleton robot, inertial measurement unit, encoders

## Abstract

Wearable exoskeleton robots have become a promising technology for supporting human motions in multiple tasks. Activity recognition in real-time provides useful information to enhance the robot’s control assistance for daily tasks. This work implements a real-time activity recognition system based on the activity signals of an inertial measurement unit (IMU) and a pair of rotary encoders integrated into the exoskeleton robot. Five deep learning models have been trained and evaluated for activity recognition. As a result, a subset of optimized deep learning models was transferred to an edge device for real-time evaluation in a continuous action environment using eight common human tasks: stand, bend, crouch, walk, sit-down, sit-up, and ascend and descend stairs. These eight robot wearer’s activities are recognized with an average accuracy of 97.35% in real-time tests, with an inference time under 10 ms and an overall latency of 0.506 s per recognition using the selected edge device.

## 1. Introduction

Wearable exoskeleton robots have recently emerged as a viable solution to assist human physical movements in various fields, such as muscular rehabilitation [1], daily activity assistance [2], and movement supports in manufacturing tasks [3,4]. Recognizing human activities would be helpful to better assist human actions via wearable robots by enhancing and customizing the robot control per activity [5]. Since exoskeletons are generally integrated by multiple IMU sensors and encoders, it is possible to implement a human activity recognition (HAR) system based on these sensors with high accuracy and low latency response [6].

HAR approaches have been widely reported with RGB-D cameras and IMU sensors via supervised machine-learning techniques. Typically, RGB-D video-based methods have been commonly applied in pose estimation [7] and activity recognition [8,9]. These computer vision-based approaches generally require multiple fields of view but cannot directly measure body movements. However, sensor-based HAR approaches can overcome the limitations of vision systems using lightweight compact and body-attached wearable sensors, measuring the body movements directly. The sensor-based HAR typically works by utilizing body-mounted sensors, smartwatches, and wristbands [10,11,12,13,14], collecting movement data directly from specific areas or positions. With these kinds of sensors, traditional machine-learning methods have been applied in HAR systems [9,15]. Subsequently, deep learning networks have become the leading solution [9] for overcoming the limitations of conventional machine learning approaches [9,10]. The deep learning-based HAR studies were mainly carried out with multiple motion sensors deployed on the chest, waist, and wrist, capturing human body activities and hand gestures [11], and achieving an overall HAR accuracy higher than 95% in [15,16,17,18,19]. However, most of these studies have been conducted using offline setups, such as PCs or laptops, thus limiting practical HAR in real-time application environments. Only a few recent studies have proposed real-time systems based on edge computing devices [20,21,22,23]. These mentioned approaches were tested using a Raspberry Pi 3 board with embedded lightweight machine classification models such as k-nearest neighbors (KNN), convolutional neural networks (CNN), and recurrent neural networks (RNN). Despite the significant use of deep learning models for activity recognition, few studies still use traditional machine-learning methods for real-time HAR environments in edge devices [24]. More than three IMU sensors were used to recognize the lower and upper limb activities such as walk, run, jog, and open doors. These studies reported a HAR accuracy of 96.28% with an overall latency of 1.325 s per recognition and an inference time of 115.18 ms in real-time tests. It should be noted that most of these studies relied on the activity signals directly measured from the body-attached sensors. Since the accuracy of HAR is highly affected by the sensor positions and deployments, it is necessary to investigate the feasibility of HAR based on the robot-mounted motion sensors.

To achieve HAR of the robot wearer’s activities, in [4], a soft wearable robot was used for HAR of industrial assembling tasks in a controlled environment. A total of 12 IMU sensors were integrated into the wearable robot. This study presented a hybrid deep learning model composed of CNN and RNN layers, achieving only 77.5% accuracy due to the complexity of the assembling tasks with an offline setup. This study utilized embedded KNN and support vector machine (SVM) models into a Raspberry Pi 3 board for real-time HAR, yielding an inference time of 5.7 ms with an overall accuracy of 98.75%. However, only four simple tasks, walk, run, stairs ascend, and stairs descend, were recognized using a thigh-mounted IMU and one force-sensitive resistor on the same ankle leg.

In this work, we have implemented a real-time HAR system with an actual wearable exoskeleton robot using integrated motion sensors, an edge device, and embedded light deep learning models. We have aimed to achieve a reasonable inference and latency time for real-time HAR. Therefore, we first tested and evaluated five deep learning models for HAR on a PC. Then, among the PC-trained models, the best ones in terms of accuracy with an inference time under 10 ms were optimized and embedded into the selected edge device. Finally, we tested and validated the performance of the optimized models in the edge device during a continuous real-time test. The main contributions of our work are as follows: We have tested the feasibility of real-time HAR with a wearable robot with integrated motion sensors and embedded light deep learning models in an edge device. Secondly, the successful results of this work demonstrate a standalone HAR system that could be used to assist human motions and tasks using wearable robots. Finally, the presented HAR approach reduces the total latency response of the prior attempts, while maintaining a recognition accuracy higher than 97% in real-time.

The subsequent sections are organized as follows: Section 2 provides a detailed description of the design of the HAR system. Section 3 describes the results achieved on the PC and an edge device. Section 4 discusses the results against prior works. Finally, Section 5 describes the main drawback, possible future works, and the conclusion of our approach.

## 2. Materials and Methods

**Figure 1:**
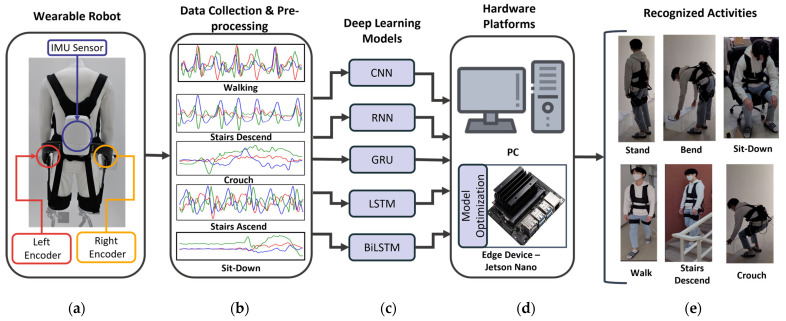
The wearable robot and implemented HAR system: (**a**) Wearable exoskeleton robot, (**b**) Data collection and preprocesses, (**c**) Deep learning models for HAR, (**d**) Hardware platforms, and (**e**) Recognized activities.

The components of the implemented real-time HAR system with a wearable robot are illustrated in Figure 1. From the left, Figure 1a shows the wearable exoskeleton robot and its embedded sensors used for data collection and HAR. Figure 1b shows samples of time series data collected from the integrated IMU in the robot backpack. The implemented deep learning models for HAR and the computing devices used are listed in Figure 1c,d, respectively. Finally, the HAR results are illustrated in Figure 1e. The following sections describe each one of these components in more detail.

### 2.1. Wearable Exoskeleton Robot and Sensors

The WEX platform is a waist-assist wearable robot developed by Hyundai Rotem. It is designed to reduce the load on the spine, prevent musculoskeletal diseases, and assist in walking or lifting heavy objects. These actions are made possible by operating the integrated motors in the same direction as the human actions to enhance muscle strength.

The wearable robot structure is carried on the shoulders and fastened at the chest, waist, and thighs by belts. The robot weighs about 6 kg, including actuators, controller units, sensors, and batteries. The assist torque is generated by a set of two 170 BLDC motors on the hip joint. Each motor has one degree of freedom (DOF) near the hip and two passive DoFs in the thigh frame symmetrically. In addition, the robot has two main kinds of sensor elements. First, two rotary encoders are inside the actuator modules in charge of measuring the angle of the hip joint. Second, one nine-axis IMU sensor, composed of a triaxial accelerometer, a triaxial gyroscope, and a triaxial magnetometer, is integrated into the robot backpack located in the back lower section of the platform. Using all the elements of the WEX platform, the robot system can have a continuous operation time of approximately 2 h.

### 2.2. Activity Data Collection

Two kinds of datasets were collected to train, test, and validate the HAR system. For both datasets, we considered the following eight activities: stand, walk, bend, crouch, stand-up, sit-down, ascend and descend stairs. The activity signals were recorded from one IMU and two rotary encoder sensors integrated into the wearable exoskeleton robot.

**Figure 2:**
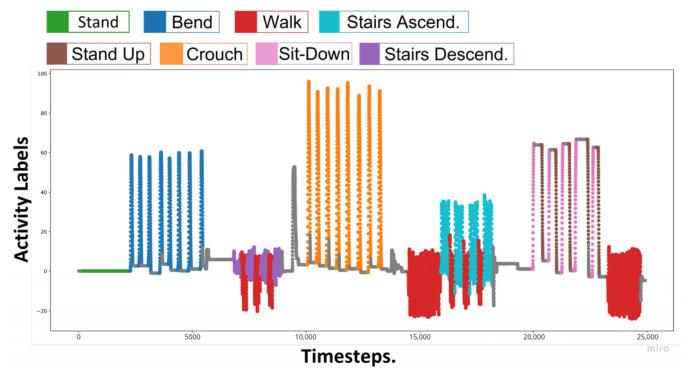
A scenario for continuous human activities of the robot wearer.

The datasets were collected according to two protocols. In the first protocol, the same activity was repeated multiple times, and the activity signals during each iteration were recorded; this record is named the epoch dataset. The epoch dataset was used to train, validate, and optimize the deep learning models on the PC and in the edge device. Meanwhile, the second protocol is illustrated in Figure 2, where the eight proposed activities were performed in a specific order to obtain a continuous activity record, naming it the continuous dataset. This continuous dataset was used to test the feasibility of HAR on the edge device with multiple actions in sequence. The data collection processes for both protocols are described in more detail in the following subsections.

#### 2.2.1. Epoch Dataset

In the epoch dataset, the signals were collected from repetitive movements per activity from four male subjects, aged between 25 and 30 years old and with heights between 1.60 and 1.80 m. Each subject performed a set of 10 repetitions for a total of 15 trials (i.e., 150 movements per activity). Once the datasets were collected for each trial, the signals were separated into epochs of three seconds with a sampling rate of 50 Hz. The number of epochs per activity was divided into stand (402), walk (818), bend (1659), crouch (1388), stand-up (706), sit-down (1701), stairs ascend (816), and stairs descend (714), totaling 8222 raw epochs for all activities.

#### 2.2.2. Continuous Activity Dataset

Each subject performed continuous activities twice in the continuous dataset according to the second protocol. During the recording procedure, the data labeling was assigned manually using physical buttons attached to the exoskeleton robot to mark the activity label on each timestep. A total of 332 epochs for 8.3 min were contained in each dataset per subject. The continuous protocol for both subjects was carried out indoors, including floors, corridors, and stairs. These continuous datasets were used to validate real-time continuous HAR with an edge device.

### 2.3. Data Preprocessing and Augmentation

For the epoch and continuous dataset, a set of preprocessing steps for sensor-based HAR were applied to clean and prepare the data for training and testing the models [9]. First, the drop-out data technique was used to clean up the incorrect data due to hardware disconnection errors during the data recording process. The same drop-out was performed for the outliers. The mean value was removed for each epoch, followed by a global normalization using the maximum and minimum values of the records to preserve the magnitude information of each activity. Consequently, a moving average filter of 5 points was applied to all the epochs for signal smoothing and de-noising of the recorded signal. This technique is selected due to its low complexity and fast execution. Then to augment the epochs, we used a sliding window overlap technique [25] to balance the epoch datasets of the eight activities. Finally, data segmentation was performed, dividing the epochs into training and validation datasets using an 80/20 ratio for five-fold tests.

### 2.4. Deep Learning Models for HAR

**Figure 3:**
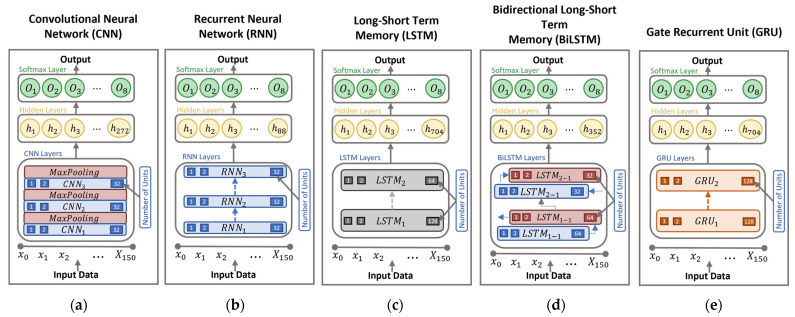
Deep Learning model structure: (**a**) CNN, (**b**) RNN, (**c**) LSTM, (**d**) BiLSTM, (**e**) GRU.

In this work, we have adopted and implemented five deep learning models for HAR: CNN, RNN, LSTM, Bi-LSTM, and GRU. These five models have shown their merits and advantages over previous sensor-based HAR works [25,26,27,28,29]. Figure 3 shows the architecture of these models. The characteristics and implementation details of the five models are given in the following subsections.

#### 2.4.1. Convolutional Neural Network

The CNN model is a neural network capable of extracting local dependencies by enforcing a sparse local connection from the input data. This model extracts features with a sliding kernel on each layer through data timesteps values. This model captures the data of unique patterns or features for each activity signal. For our application of HAR, a one-dimensional variant was selected, since this model could extract features at a low computational cost.

Our implemented CNN model, named CNN-3L, is shown in Figure 3a. This model comprises one input layer with a length of 150 timesteps, followed by three CNN layers of 32 units with a kernel size of three and a rectifier linear unit (ReLU) as an activation function per layer. After each block, a max pooling layer with a pool size of two is applied to reduce the number of trainable parameters and control overfitting. Finally, a dense layer with 272 hidden units is added in conjunction with a SoftMax layer with eight output neurons.

#### 2.4.2. Vanilla Recurrent Neural Network

The RNN model is a basic framework applied in natural language processing (NLP) or speech recognition problems due to its capability of extracting the features and patterns of sequential activity signals. Unlike feed-forward neural networks, the RNN model processes the data in a recurrent form using the hidden states, commonly referred to as memory components, on each node to retain sequential information from the past input data. This model presents a lower computational cost during training by sharing the weight values across the data timesteps. Regarding the improvement, against CNN models in time-series data, this model can handle an arbitrary input/output length, making it feasible for prediction applications based on prior data information.

Our implemented RNN model, named RNN-2L, is presented in Figure 3b. It is composed of a total of two RNN layers with 32 units and ReLU activation functions. Then, it is followed by a dense layer with 88 hidden units and a SoftMax layer with eight output neurons.

#### 2.4.3. Long-Short-Term Memory

The long short-term memory (LSTM) model is an enhanced version of RNN. It can overcome the vanishing gradient problem since it can retain feature information for a longer time. The model uses a mechanism comprising three gates, namely forget, input, and output gates. These structures allow the model to choose which information is stored and which gets forgotten, saving the long-term dependence in the context state. This process starts with the forgotten gate using the hidden state of the last state and the current input value to decide which relevant information is kept for the current LSTM cell. Then the input gate determines which new data can be added from the current time step. The new context state is updated with the result of these two gates. Finally, the output value is obtained between the initial context state and the current input, to create a new hidden and context state to use in the next LSTM model.

Our LSTM model, named LSTM-2L, is presented in Figure 3c, in which a total of two LSTM layers with 128 and 64 units were implemented using a ReLU activation function. Then, a fully connected dense layer is used with 704 units and one SoftMax layer, with eight output neurons.

#### 2.4.4. Bidirectional Long-Short-Term Memory

The bidirectional long short-term memory (Bi-LSTM) model allows the use of an input flow in two directions, backward and forward, unlike the baseline LSTM model, which only admits one single direction. This model can extract features relevant to the future and past time steps.

Our Bi-LSTM model, named Bi-LSTM-2L, is presented in Figure 3d. The model is composed of two Bi-LSTM layers with 64 and 32 units, and a ReLU activation function. Then, it is followed by a dense layer with 352 neurons and one SoftMax layer with eight output neurons.

#### 2.4.5. Gate Recurrent Unit

The gate recurrent unit (GRU) is a compact neuronal network version of LSTM that removes the context state. The GRU model only uses the hidden state to pass the prior relevant information. This model is used to retain the memory capability in a compact form, reducing the number of tensor operations and making the model faster to train.

Our implemented GRU model, named GRU-2L, is shown in Figure 3e, where a total of two GRU layers with 128 units and a ReLU activation function are used. The model is followed by a fully connected dense layer with 704 neurons and one SoftMax with eight output neurons.

### 2.5. HAR Training and Evaluation on PC

The training and validating processes were carried out using the epoch datasets of the four subjects on a PC. For this process, a total of 892,839 training epochs and 224,209 validating epochs were used. For the training process, a PC with an Nvidia RTX 2070 GPU of 8 Gb memory was used with a learning rate of 0.0003 and a batch size of 64 for each model. All the models were written using Python 3.8 with TensorFlow and Keras. To evaluate the performance of the implemented five deep learning models, two conventional criterion metrics were used: accuracy and inference time. To calculate the accuracy, Equation (1) was used, where $T_{p},\;F_{n},\;F_{p}$ and $T_{n}$ represents the sample number of true positives, false negatives, and true negatives, respectively. On the other hand, the inference time $t_{inference}\left(ms\right)$ represents the time needed for the model to output a classification label. Meanwhile, the inference time is given as Equation (2), where $t_{inp}$ is the time value when the data is input to the model. $t_{out}$ is the time value when the result classification label is obtained.

(1) $$Accuracy\;\left(\%\right)=\frac{T_{p}+T_{n}}{T_{p}+T_{n}+F_{p}+F_{n}}x\;100$$

(2) $$t_{inference}\left(\mathrm{ms}\right)=t_{out}-t_{inp}$$

### 2.6. HAR Training and Evaluation on Edge Device

After the HAR classifiers were trained and validated on the PC, the best models in terms of inference time and accuracy were transferred into our edge computing device. For the edge computing of HAR, we selected Nvidia Jetson Nano, among other edge devices, due to its capacity for training, optimizing, and testing the implemented models on the edge device. This single-board computer is capable of these tasks via the integrated quad-core ARM Cortex-A57 CPU, a dedicated Nvidia graphics card 128-core Maxwell, with a 4 Gb of RAM, and an ARM operating system based on Ubuntu 18.04. Furthermore, due to the compatibility with multiple deep learning libraries such as TensorFlow, Python, and TensorRT, the models implemented were optimized, decreasing the computational cost and inference time for each classification. After testing the five models on PC, the best models were selected and optimized, based on an accuracy higher than 95% and an inference time under 10 ms. This model optimization was carried out using the trained models in the TF-TensorRT engine. This framework reduces the precision range used on each layer by decreasing the number of decimal digits used on each mathematical operation of FP32 to FP16. To validate the performance of the HAR models in the edge device, we compared the performance of the selected models from the PC. Finally, we tested the real-time results of eight activities with the optimized model.

## 3. Results

The following sections present the results from the tests on PC and edge device, and finally, the real-time online tests. We compared and validated the accuracy using the epoch dataset and computed the inference time to determine the best models for the edge device. Then three selected models were embedded in the Jetson Nano device, validating the performance of these models via real-time tests.

### 3.1. HAR Results on PC

**Figure 4:**
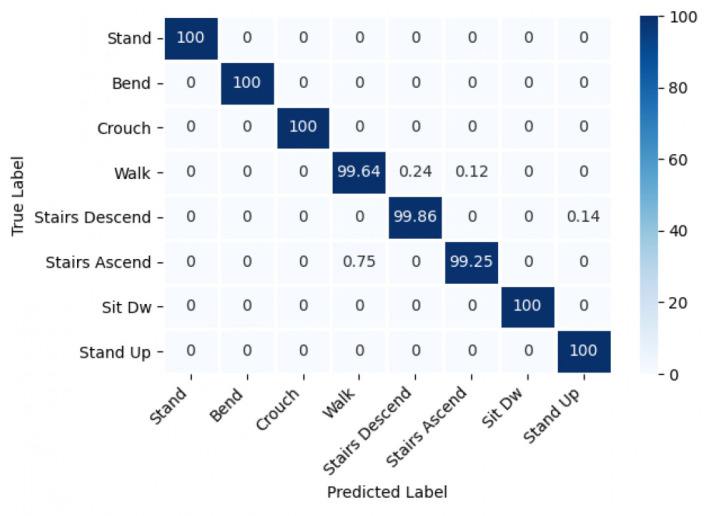
A sample confusion matrix with the Bi-LSTM-2L model and epoch dataset.

For the results shown below, the epoch dataset was used to evaluate the performance of the five deep network models on the PC. Table 1 shows the average accuracy of each model and the accuracy for individual activities where the Bi-LSTM-2L model achieved the best performance. The corresponding confusion matrix is presented in Figure 4 for all eight activities. In this case, all the deep learning models achieved a high accuracy of over 98%, although some confusion was noticed among locomotion activities such as walk, stairs ascend, and stairs descend. Table 2 shows the accuracy of HAR for four different subjects with the Bi-LSTM-2L model.

### 3.2. HAR Results on Edge Devices

After the PC test, the TensorRT engine was used to embed the best models with an inference time under 10 ms into the edge device. Based on the preliminary tests, the BiLSTM-2L and GRU-2L models produced an inference time higher than 20 ms, which is beyond our acceptance criteria. Therefore, these models were dropped for the continuous HAR test with the edge device. On the other hand, for the RNN-2L and LSTM-2L models, we reduced the number of layers from two to one to decrease their inference time, ending up with the new models, namely, RNN-1L and LSTM-1L. Finally, to evaluate the performance of the edge device, we tested the following five models (i.e., CNN-3L, RNN-2L, RNN-1L, LSTM-2L, and LSTM-1L). The information regarding model sizes, inference times, and overall accuracies for the two subjects is summarized in Table 3. We noticed that the accuracy values compared to the non-optimized models do not present a significant difference. Meanwhile, a decrease in the inference time is notable, reducing it by 56% with CNN-3L, 80% RNN-2L, 78% RNN-1L, 27% LSTM-2L, and 25% LSTM-1L, compared to the non-optimized models with TensorRT. From the results of Table 3, LSTM-1L, LSTM-2L, and RNN-2L models are excluded due to their prolonged inference time, resulting in only two models (i.e., CNN-3L, RNN-1) for real-time HAR.

### 3.3. Real-Time Continuous HAR

**Figure 5:**
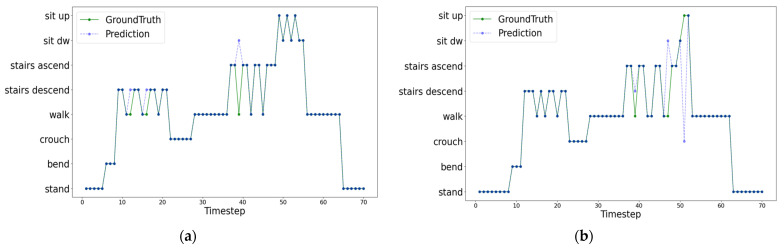
Continuous real-time HAR results from two subjects: (**a**) S_1_ and (**b**) S_2_.

We tested the HAR system with the continuous activity dataset for real-time evaluations of the implemented and optimized HAR system, including the wearable robot, integrated sensors, and edge device. Table 4 shows the accuracy and inference time of the CNN-3L and RNN-1L models using the continuous dataset for two subjects. Overall, the accuracies are slightly lower than the results from the epoch dataset. Of the five deep learning models tested, CNN-3L produced the best performance as it achieved the lowest inference time of 4.97 ms without a significant loss of accuracy. Figure 5 shows the continuous HAR results against the ground truth activity labels with the CNN-3L model. The HAR results present an accurate classification, but some misrecognition of the transition between activities (i.e., when activity transits one from another) is noticed. The activity recognition was performed every 0.5 s, and each recognition was performed with a latency time of 0.506 s, including preprocessing and inference time.

**Figure 6:**
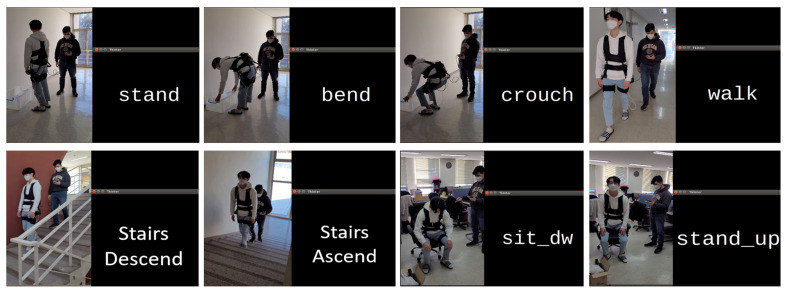
Recognition examples and screenshots with HAR system results during continuous real-time tests.

Finally, Figure 6 shows some sample recognition results of the performed activities, and the screenshots of the output label for the current activity displayed on the screen of the edge device from the real-time online tests of the whole integrated HAR system. A recognition accuracy above 95% was achieved with an inference time under 10 ms with CNN-3L.

## 4. Discussion

In this paper, we have performed real-time HAR of the exoskeleton wearer’s activities, using the integrated sensors of the wearable robot. The proposed HAR system has been implemented, tested, and validated using the proposed deep learning models on the edge device. First, we trained and tested the deep learning models on a PC where the Bi-LSTM-2L model achieves the highest accuracy of 99.79%, with the epoch dataset among the five models proposed without considering the inference time needed. Meanwhile, for the classifier models, the CNN-3L model was selected, optimized, and embedded in the Jetson Nano as the best model, achieving an average accuracy of 97.35% with an inference time of 4.97 ms and obtaining a general latency of 0.506 s in the real-time tests.

Recent HAR studies based on edge devices [20,21,22,23], have used a Raspberry Pi 3 board to implement SVM, custom CNNs, and GRU models in real-time tests. In all these cases, a minimum of three IMU sensors were necessarily placed on different body parts, such as the neck, wrists, waist, or ankles. Due to the multiple feature channels, the processing time for collection and inference was prolonged, reaching a recognition accuracy above 96.28% with an inference time of 115.18 ms and an overall latency time higher than 1.32 s. In contrast to these approaches, we have used a Jetson Nano board as an edge device to embed, train, and optimize the deep learning HAR classifiers on it. In our approach, only one IMU sensor and two rotary encoders were used to achieve the high recognition accuracy of the eight activities. Our work achieved a latency time of 0.506 s, the shortest compared to the previous studies’ time. These results demonstrate that real-time HAR could be performed for the wearable robot using a standalone system. Among the mentioned HAR works based on edge devices, the best approach was recently addressed in [24]. In this study, a HAR approach was reported based on a Raspberry Pi 3 board as an application for a wearable robot or leg prosthesis. The traditional machine learning models KNN and SVM used in this attempt achieve an overall accuracy of 99.41% and a latency window of 0.566 s using a single 9-axis IMU and one resistor force sensor. Although the minimal difference in latency was 60 ms between this approach and our proposal, only four simple locomotion tasks, walk, stand, stairs ascend, and descend, were recognized using traditional machine learning approaches. In addition, this work used the body-attached sensors instead of the robot-mounted sensors.

Despite the previously mentioned HAR studies based on edge devices, few approaches link this structure with wearable exoskeleton robots. An instance of this lack is presented in [4], where an actual exoskeleton robot is used for offline HAR without tests on real-time environments with edge devices. In this study, an accuracy of 77.5% was achieved recognizing complex assembling tasks. For this, a hybrid CNN-RNN model was used with twelve six-axis IMU sensors distributed in different locations, such as the head, forearms, thighs, wrists, and ankles. Contrary to this approach, our work aims to provide a HAR system, based on exoskeletons and edge devices, to create a standalone system capable of being used in real-time tests with higher accuracy and a lower latency response.

## 5. Conclusions

The main practical applications of this study are related to the use of an exoskeleton robot to assist human motions. For instance, by recognizing current human activities, the wearable robot could reduce the workload of carrying or lifting heavy objects, and prevent musculoskeletal diseases by improving the user’s muscle strength in the rehabilitation process. The present HAR system has one drawback, which is the misrecognition of the transition activities. This problem could be solved by modeling and training these activities in deep learning models. Furthermore, it is possible to deploy more sensors in the wearable robot for more complex or intricate tasks and extending our models to recognize them.

To summarize, we have validated and confirmed the feasibility of real-time HAR with the wearable exoskeleton robot and HAR system. The presented results demonstrate that it is possible to achieve real-time HAR of the robot wearer’s eight activities. In real-time tests, we have achieved an overall accuracy of 97.35%, with an inference time under 10 ms using the Jetson Nano board as an edge device with deep learning classifiers based on integrated sensors.

## Figures and Tables

**Table 1:** The accuracies of HAR from the five deep learning models tested on PC.

Activity	Model Accuracy (%)				
	CNN-3L	RNN-2L	LSTM-2L	Bi-LSTM-2L	GRU-2L
Stand	100	100	100	100	100
Bend	100	98.19	99.86	100	100
Crouch	100	97.08	100	100	99.70
Walk	100	96.37	99.05	99.64	99.34
Stairs Descend	98.92	97.43	99.71	99.86	98.85
Stairs Ascend	100	98.49	98.63	99.25	99.84
Sit-Down	97.29	99.87	100	100	99.93
Stand-Up	100	99.86	100	100	100
Average	99.53	98.41	99.34	99.84	99.83

**Table 2:** HAR accuracies with the Bi-LSTM-2L and epoch dataset from four subjects.

Activity	Bi-LSMT-2L Accuracy per Subject			
	S_1_	S_2_	S_3_	S_4_
Stand	100	100	100	100
Bend	100	100	99.91	100
Crouch	100	100	99.57	98.71
Walk	99.64	99.52	100	100
Stairs Descend	99.86	99.71	100	100
Stairs Ascend	99.25	99.13	100	100
Sit-Down	100	100	100	100
Stand-Up	100	100	99.19	99.04
Average	99.84	99.80	99.83	99.72

**Table 3:** HAR accuracies, model sizes, and inference time for two subjects obtained with the Jetson Nano board and epoch dataset.

Model	Model Size (KB)	Inference Time (ms)	Accuracy (%)	
			S_1_	S_2_
CNN-3L	231.6	4.97	98.96	99.41
RNN-2L	616.5	15.2	97.53	98.46
RNN-1L	428.1	8.8	97.30	97.83
LSTM-2L	823.7	19.6	99.34	99.52
LSTM-1L	515.1	14.4	98.51	99.24

**Table 4:** HAR accuracies and inference time with the Jetson Nano board and continuous dataset.

Subject (Model)	Accuracy (%)	Inference Time (ms)
S_1_ (CNN-3L)	97.56	4.97
S_1_ (RNN-1L)	86.19	8.47
S_2_ (CNN-3L)	97.15	4.98
S_2_ (RNN-1L)	89.31	8.7

## Data Availability

Not applicable.

## References

[1] Huo W., Mohammed S., Moreno J.C., Amirat Y. (2016). Lower Limb Wearable Robots for Assistance and Rehabilitation: A State of the Art. IEEE Syst. J..

[2] Chen K., Zhang D., Yao L., Guo B., Yu Z., Liu Y. (2021). Deep Learning for Sensor-Based Human Activity Recognition: Overview, Challenges, and Opportunities. ACM Comput. Surv..

[3] de Looze M.P., Bosch T., Krause F., Stadler K.S., O’Sullivan L.W. (2016). Exoskeletons for Industrial Application and Their Potential Effects on Physical Work Load. Ergonomics.

[4] Kuschan J., Burgdorff M., Filaretov H., Krüger J. (2021). Inertial Measurement Unit Based Human Action Recognition for Soft-Robotic Exoskeleton. IOP Conference Series: Materials Science and Engineering.

[5] Tucker M.R., Olivier J., Pagel A., Bleuler H., Bouri M., Lambercy O., Millán J.D.R., Riener R., Vallery H., Gassert R. (2015). Control Strategies for Active Lower Extremity Prosthetics and Orthotics: A Review. J. Neuroeng. Rehabil..

[6] Schmidt P., Reiss A., Hanselmann M., Stiefelhagen R. CNN-Based Sensor Fusion Techniques for Multimodal Human Activity Recognition. Proceedings of the 2017 ACM International Symposium on Wearable Computers.

[7] Park S., Ji M., Chun J. (2018). 2D Human Pose Estimation Based on Object Detection Using RGB-D Information. KSII Trans. Internet Inf. Syst..

[8] Zhang X., Xu C., Tian X., Tao D. (2020). Graph Edge Convolutional Neural Networks for Skeleton-Based Action Recognition. IEEE Trans. Neural. Netw. Learn. Syst..

[9] Minh Dang L., Min K., Wang H., Jalil Piran M., Hee Lee C., Moon H. (2020). Sensor-Based and Vision-Based Human Activity Recognition: A Comprehensive Survey. Pattern Recognit..

[10] Wang J., Chen Y., Hao S., Peng X., Hu L. (2019). Deep Learning for Sensor-Based Activity Recognition: A Survey. Pattern Recognit. Lett..

[11] Rivera P., Valarezo E., Kim T.S. (2021). An Integrated ARMA-Based Deep Autoencoder and GRU Classifier System for Enhanced Recognition of Daily Hand Activities. Int. J. Pattern Recognit. Artif. Intell..

[12] Lee M.W., Khan A.M., Kim T.S. (2011). A Single Tri-Axial Accelerometer-Based Real-Time Personal Life Log System Capable of Human Activity Recognition and Exercise Information Generation. Pers. Ubiquitous Comput..

[13] Khan A.M., Lee Y.K., Lee S., Kim T.S. (2010). Accelerometer’s Position Independent Physical Activity Recognition System for Long-Term Activity Monitoring in the Elderly. Med. Biol. Eng. Comput..

[14] Khan A.M., Lee Y.K., Lee S.Y., Kim T.S. (2010). A Triaxial Accelerometer-Based Physical-Activity Recognition via Augmented-Signal Features and a Hierarchical Recognizer. IEEE Trans. Inf. Technol. Biomed..

[15] Ramanujam E., Perumal T., Padmavathi S. (2021). Human Activity Recognition with Smartphone and Wearable Sensors Using Deep Learning Techniques: A Review. IEEE Sens. J..

[16] Kim Y.W., Joa K.L., Jeong H.Y., Lee S. (2021). Wearable Imu-Based Human Activity Recognition Algorithm for Clinical Balance Assessment Using 1d-Cnn and Gru Ensemble Model. Sensors.

[17] Jantawong P., Hnoohom N., Jitpattanakul A., Mekruksavanich S. A Lightweight Deep Learning Network for Sensor-Based Human Activity Recognition Using IMU Sensors of a Low-Power Wearable Device. Proceedings of the ICSEC 2021—25th International Computer Science and Engineering Conference.

[18] Sherratt F., Plummer A., Iravani P. (2021). Understanding Lstm Network Behaviour of Imu-Based Locomotion Mode Recognition for Applications in Prostheses and Wearables. Sensors.

[19] Fu Z., He X., Wang E., Huo J., Huang J., Wu D. (2021). Personalized Human Activity Recognition Based on Integrated Wearable Sensor and Transfer Learning. Sensors.

[20] Mascret Q., Bielmann M., Fall C.-L., Bouyer L.J., Gosselin B. Real-Time Human Physical Activity Recognition with Low Latency Prediction Feedback Using Raw IMU Data. Proceedings of the 2018 40th Annual International Conference of the IEEE Engineering in Medicine and Biology Society (EMBC).

[21] Tang Y., Zhang L., Wu H., He J., Song A. (2022). Dual-Branch Interactive Networks on Multichannel Time Series for Human Activity Recognition. IEEE J. Biomed. Health Inf..

[22] Yeh Y.H., Wong D.P.Y., Lee C.T., Chou P.H. (2022). Deep Learning-Based Real-Time Activity Recognition with Multiple Inertial Sensors. Proceedings of the ACM International Conference Proceeding Series.

[23] Wang X., Zhang L., Huang W., Wang S., Wu H., He J., Song A. (2022). Deep Convolutional Networks with Tunable Speed-Accuracy Tradeoff for Human Activity Recognition Using Wearables. IEEE Trans. Instrum. Meas..

[24] Cheng S., Bolivar-Nieto E., Gregg R.D. (2021). Real-Time Activity Recognition with Instantaneous Characteristic Features of Thigh Kinematics. IEEE Trans. Neural Syst. Rehabil. Eng..

[25] le Guennec A., Malinowski S., Tavenard R. Data Augmentation for Time Series Classification Using Convolutional Neural Networks. Proceedings of the ECML/PKDD Workshop on Advanced Analytics and Learning on Temporal Data.

[26] Banjarey K., Prakash Sahu S., Kumar Dewangan D. A Survey on Human Activity Recognition Using Sensors and Deep Learning Methods. Proceedings of the 2021 5th International Conference on Computing Methodologies and Communication (ICCMC).

[27] Sansano E., Montoliu R., Belmonte Fernández Ó. (2020). A Study of Deep Neural Networks for Human Activity Recognition. Comput. Intell..

[28] Zhang S., Li Y., Zhang S., Shahabi F., Xia S., Deng Y., Alshurafa N. (2022). Deep Learning in Human Activity Recognition with Wearable Sensors: A Review on Advances. Sensors.

[29] Bozkurt F. (2022). A Comparative Study on Classifying Human Activities Using Classical Machine and Deep Learning Methods. Arab. J. Sci. Eng..

